# Digital Life Coaching During Stem Cell Transplantation: Development and Usability Study

**DOI:** 10.2196/33701

**Published:** 2022-03-04

**Authors:** Rahul Banerjee, Chiung-Yu Huang, Lisa Dunn, Jennifer Knoche, Chloe Ryan, Kelly Brassil, Lindsey Jackson, Dhiren Patel, Mimi Lo, Shagun Arora, Sandy W Wong, Jeffrey Wolf, Thomas Martin III, Anand Dhruva, Nina Shah

**Affiliations:** 1 Division of Hematology/Oncology Department of Medicine University of California San Francisco San Francisco, CA United States; 2 Department of Epidemiology and Biostatistics University of California San Francisco San Francisco, CA United States; 3 Pack Health Birmingham, AL United States; 4 Division of Hematology/Oncology Department of Pharmacy University of California San Francisco San Francisco, CA United States

**Keywords:** digital health, life coaching, multiple myeloma, stem cell transplantation, stem cell therapy, cancer, high-dose chemotherapy, patient engagement, feasibility, digital life coaching, mobile phone

## Abstract

**Background:**

For patients with multiple myeloma receiving high-dose chemotherapy followed by autologous stem cell transplantation (SCT), acute life disruptions and symptom burden may lead to worsened quality of life (QOL) and increased emotional distress. Digital life coaching (DLC), whereby trained coaches deliver personalized well-being–related support via phone calls and SMS text messaging, has been shown to improve QOL among SCT survivors. However, DLC has not been investigated during the acute peri-SCT period, which is generally characterized by symptomatic exacerbations and 2-week hospitalizations.

**Objective:**

We launched a single-arm pilot study to investigate the feasibility of patient engagement with DLC during this intensive period.

**Methods:**

We approached English-speaking adult patients with multiple myeloma undergoing autologous SCT at our center. Enrolled patients received 16 weeks of virtual access to a life coach beginning on day −5 before SCT. Coaches used structured frameworks to help patients identify and overcome personal barriers to well-being. Patients chose the coaching topics and preferred communication styles. Our primary endpoint was ongoing DLC engagement, defined as bidirectional conversations occurring at least once every 4 weeks during the study period. Secondary endpoints were electronic patient-reported outcome assessments of QOL, distress, and sleep disturbances.

**Results:**

Of the 20 patients who were screened, 17 (85%) chose to enroll and 15 (75%) underwent SCT as planned. Of these 15 patients (median age 65 years, range 50-81 years), 11 (73%) demonstrated ongoing DLC engagement. The median frequency of bidirectional conversations during the 3-month study period was once every 6.2 days (range 3.9-28 days). During index hospitalizations with median lengths of stay of 16 days (range 14-31 days), the median frequency of conversations was once every 5.3 days (range 2.7-15 days). Electronic patient-reported outcome assessments (94% adherence) demonstrated an expected QOL nadir during the second week after SCT. The prevalence of elevated distress was highest immediately before and after SCT, with 69% of patients exhibiting elevated distress on day −5 and on day +2.

**Conclusions:**

DLC may be feasible for older patients during intensive hospital-based cancer treatments such as autologous SCT for multiple myeloma. The limitations of our study include small sample size, selection bias among enrolled patients, and heterogeneity in DLC use. Based on the positive results of this pilot study, a larger phase 2 randomized study of DLC during SCT is underway to investigate the efficacy of DLC with regard to patient well-being.

**Trial Registration:**

ClinicalTrials.gov NCT04432818; https://clinicaltrials.gov/ct2/show/NCT04432818.

## Introduction

Multiple myeloma (MM) is an incurable hematologic malignancy in older adults. Unlike in many other malignancies, upfront use of myeloablative chemotherapy followed by autologous stem cell transplantation (SCT) remains the standard of care for MM in eligible patients [1,2]. Autologous SCT is marked by acute symptomatic toxicities during the first 100 days after transplantation, such as fatigue, pain, and anorexia [3-6]. Sudden functional limitations, increased isolation, and nonrestful inpatient environments may also contribute to emotional distress. Patients must be monitored closely for other post-SCT toxicities and may, on average, spend over 30% of their days during this 3-month period either hospitalized or at clinic appointments [7,8]. Furthermore, most patients with MM who are employed prior to undergoing SCT are unable to return to work thereafter [9]. These factors, in turn, may lead to significant personal costs from transportation-related expenses and missed workplace productivity.

Specific manifestations of peri-SCT life disruptions may include worsened quality of life (QOL), elevated anxiety or emotional distress, and worsened sleep disturbances. These symptoms are particularly relevant for patients with MM who tend to be older and may have poorer baseline QOL than patients with other malignancies [10]. Previous studies, while limited by substantial heterogeneity in patient populations and survey-based inventories, suggest that well-being reaches its nadir 1-2 weeks after SCT before recovering in subsequent months [5,11-16]. Even so, long-term consequences of these short-term exacerbations may include persistent decreases in QOL, lowered posttransplant medication adherence, psychological comorbidities, reliance on potentially inappropriate medications such as benzodiazepines, and increased risk of hospital readmissions [17-23]. Several hospital-based interventions have thus been studied to target well-being during SCT, such as scheduled palliative care consultations, structured exercise programs, acupuncture, music therapy, and programmed room lighting [24-29]. However, these strategies may be limited by in-person provider availability in the inpatient setting or the need for “extra” clinic appointments in the outpatient setting.

Digital life coaching (DLC), whereby patients receive well-being−related support from trained coaches via bidirectional phone calls and SMS text messaging on their personal phones, may be able to address these limitations because of its virtual and location-agnostic nature. Life coaching, whereby trained coaches provide support and longitudinal accountability to empower patients to set and accomplish personal goals, has been effective in several ambulatory cancer populations [30-39]. DLC can reach patients both during and after their index SCT hospitalizations, which is a priority for patient-facing digital tools in this population [40]. While feasible among SCT survivors [41,42], DLC-type interventions have not been studied during the acute 100-day period immediately preceding and following SCT. Given the paucity of studies on the use of communication-based digital technologies among older adults [43], it is similarly unclear how acceptable DLC can be for patients with MM. We thus launched a pilot study to assess the feasibility of a DLC program for patients with MM actively undergoing SCT.

## Methods

### Study Design and Intervention

We launched a single-arm, pilot feasibility study of DLC among adult patients with MM undergoing nontandem autologous SCT at our center. English proficiency and mobile phone ownership were required for study participation; however, neither smartphone ownership nor mobile app installation were needed. There were no restrictions on time frames for pre-SCT stem cell collection or on agents used for stem cell mobilization. Based on a 68% rate of engagement in a prior study of DLC among SCT survivors and an assumption that DLC engagement below 33% would not merit further study [41], we enrolled 15 patients to exclude the possibility (with 1-sided α .05 and 90% power) of DLC engagement falling below this threshold. Patients who enrolled in the study but did not ultimately undergo autologous SCT were replaced. All patients provided informed consent prior to study enrollment. This study was approved by the University of California San Francisco Institutional Review Board (ClinicalTrials.gov NCT04432818).

Regarding the DLC platform itself, 2 life coaches employed by Pack Health [44] were paired with all patients enrolled in this study. Both coaches were certified by the National Board for Health and Wellness Coaching [45]. Coaches reached out to patients to coordinate their first coaching call beginning no earlier than day −5 before SCT. Patients then received 16 weeks of free unlimited access to their life coach by phone, SMS text messaging, or email. Coaches used structured frameworks longitudinally, including the Transtheoretical Model, Fogg Behavior Model, SMART (Specific, Measurable, Achievable, Relevant, and Time-based) Framework, and Pathways Thinking, to help patients identify and overcome personal barriers to well-being [46-53]. The content of the coaching curriculum was highly personalized to each patient at any given time and was not standardized per the study protocol. However, coaches did attempt to discuss several components of wellness (physical health, mental health, nutrition, exercise, sleep, and financial health) at least once during the study period. Coaches were not medical providers and were not licensed to address medical or psychiatric issues; as such, coaches were instructed to refer patients back to their SCT providers with any clinical concerns.

Patients could communicate bidirectionally with their coach by SMS text messaging, phone call, or email. This contact information was standardized for each coach; in other words, patients could add their coach to their list of phone contacts and communicate with them as they would communicate with loved ones. While coaches encouraged the use of weekly check-in phone calls, the actual cadence and communication methods of coaching were personalized to each patient based on their individual goals and preferences. Caregivers were allowed to join or participate in coaching as well, although all content was specifically geared toward patients themselves. Coaches organized phone calls and responded to messages during business hours for the DLC vendor (8 AM to 5 PM CST, corresponding to GMT −6); all enrolled patients were on PST (GMT −8). If patients did not respond to messages from their coaches, follow-up messages were sent no more frequently than 3 times per week. Patients were not contacted by coaches after the conclusion of the study period.

### Data Collection

The primary objective of this study was to assess the rate of ongoing patient engagement with the DLC program using an intent-to-treat approach during the 16-week study period. We adopted the definition of feasibility used in a previous study of DLC among SCT survivors—patient-initiated engagement at least once with the DLC platform in at least 68% of patients over a 3-month period [41]—with 2 a priori modifications. First, we focused on bidirectional conversations (including phone calls lasting at least 1 minute) as examples of meaningful engagement even if initiated by the coach. Second, we adopted a stricter definition of feasibility as at least one bidirectional conversation every 4 weeks during the 16-week period rather than at least once over 3 months. Our rationale for this second modification was to assess the practicality of *ongoing* patient-coach conversations across a dynamic 100-day period including index hospitalizations as well as the initiation of post-SCT maintenance therapy.

The secondary objectives of this study were to explore the results of email-based electronic patient-reported outcome (ePRO) assessments measuring QOL, emotional distress, and sleep disturbances. ePROs were assessed in accordance with the Checklist for Reporting Results of Internet E-Surveys [54]. To measure QOL, we administered the 10-item Patient-Reported Outcome Measurement Information System (PROMIS) Global Health Scale instrument v1.2. To measure emotional distress, we administered the single-item National Comprehensive Cancer Network (NCCN) Distress Thermometer excluding the problem list. To measure sleep disturbances, we administered the 4-item PROMIS Sleep Disturbance Short Form 4a. These inventories, comprising 15 questions across 4 pages including a welcome page, were assessed through a secure web-based Research Electronic Data Capture (REDCap; Vanderbilt University) platform hosted on the study site’s server. Patients received unique weblinks to complete ePRO assessments through emails sent from REDCap to their personal email addresses. These emails were sent at 12 discrete timepoints: weekly for the first 8 weeks of the study (day −5 to day +50) and then biweekly thereafter (day +51 to day +106). No ePRO questions were mandatory, no incentives were provided for ePRO assessment completion, and responses were not editable or rewritable after completion of each timepoint. The results of ePRO assessments were not shared with patients’ coaches or clinical teams at any time.

### Data Analysis and Statistical Considerations

For our primary endpoint of ongoing DLC engagement, we calculated SCT-relative dates for each bidirectional conversation within each of four 4-week study subperiods: day −5 to +22, day +23 to +50, day +51 to +78, and day +79 to +106. Based on our sample size of 15 patients, we defined feasibility as the presence of ongoing engagement in 10 or more patients (ie, at least 68% of patients) based on a previous study of DLC among SCT survivors [41].

For our secondary objectives involving ePRO assessments, we converted raw PROMIS inventory scores into T-scores to reflect a reference population with a mean score of 50 and SD of 10 as per their respective scoring manuals. We analyzed PROMIS inventory scores and Distress Thermometer scores at each timepoint descriptively using medians and ranges. Based on previous studies of patients with cancer, we defined clinically meaningful changes in PROMIS instruments as an increase or decrease of 5 or more points [55-57]. Specifically, we compared median T-scores at each timepoint to their baseline values. We defined worsened physical or mental QOL as a decrease of 5 or more points and worsened sleep as an increase of 5 or more points. We separately calculated the percentages of patients at each ePRO timepoint with elevated distress, defined as a Distress Thermometer score of 4 or higher [58].

Given our small sample size, we did not perform any longitudinal modeling of ePRO data. All analyses were performed using Stata (StataCorp) and R version 4.0.2 (R Foundation for Statistical Computing).

## Results

### Enrollment and Baseline Characteristics

**Figure 1:**
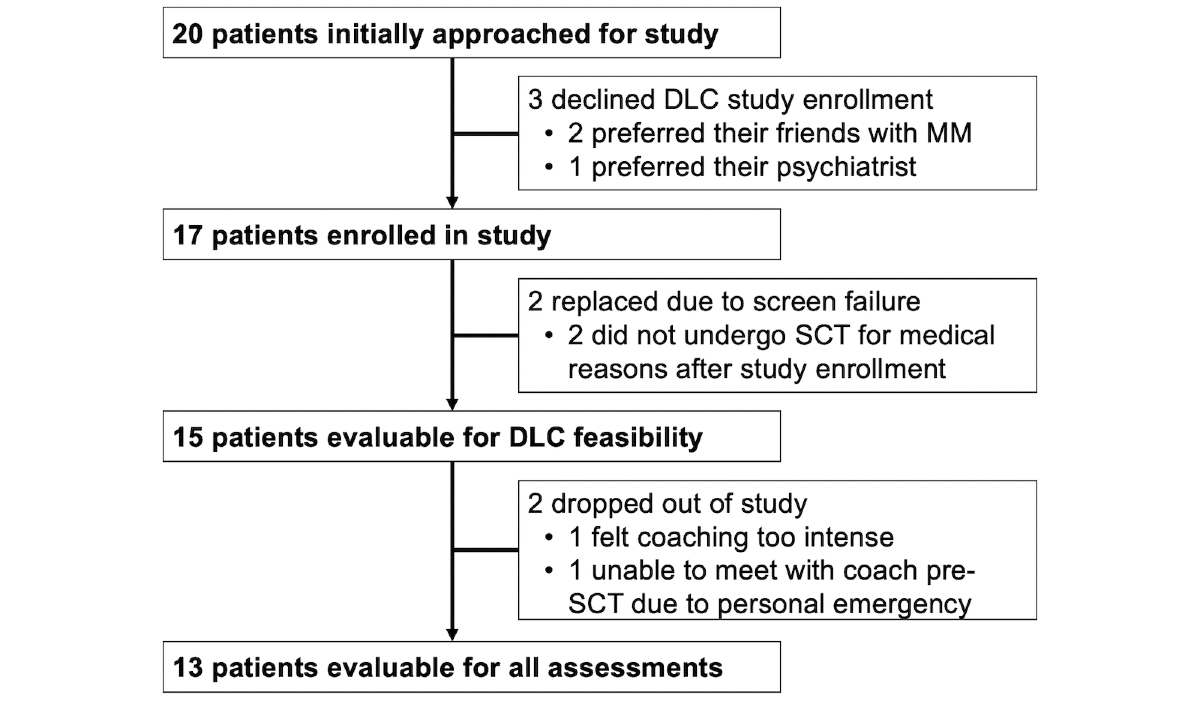
Flow diagram of enrolled patients. Abbreviations: DLC, digital life coaching; MM, multiple myeloma; SCT, stem cell transplantation.

Of the 20 patients approached between June 2020 and November 2020, 17 (85%) enrolled as outlined in Figure 1. However, 2 of these 17 (12%) patients did not undergo planned SCT for medical reasons and thus were replaced before DLC initiation. Of the 15/20 (75%) remaining patients, 2 (13%) dropped out of the study before coaching was initiated: one because she felt that the planned frequency of coaching would become too intense during SCT hospitalization and another because of a personal emergency (wildfire-related evacuations) that prevented her from coordinating a time to speak with her coach before arriving to the hospital to undergo SCT. Thus, 13/15 (87%) patients completed ePRO assessments beyond the baseline assessment and were evaluable for our secondary endpoints of QOL, emotional distress, and sleep disturbances.

Baseline characteristics of the 15 patients evaluable for DLC feasibility are summarized in Table 1. The median age of the enrolled patients was 65 years (range 50-81 years); 4/15 (27%) patients were 70 years or older at the time of SCT. The median time between MM diagnosis and SCT was 7 months (range 4-108 months). A total of 4/15 patients (27%) had an Eastern Cooperative Oncology Group performance status of 1; the remainder had a performance status of 0. Most patients (12/15, 80%) received full-dose melphalan conditioning (200 mg/m^2^) prior to SCT. All patients were hospitalized for SCT, with a median length of stay of 16 days (range 14-31 days).

**Table 1:** Baseline characteristics of evaluable patients (N=15).

Characteristic		Value, n (%)
**Age at the time of SCT^a^** **(years)**		
	50-59.9	3 (20)
	60-69.9	8 (53)
	70-79.9	3 (20)
	≥80	1 (7)
**Gender**		
	Male	7 (47)
	Female	8 (53)
**Time since diagnosis (months)**		
	0-11.9	11 (73)
	≥12	4 (27)
**Performance status**		
	ECOG PS^b^ 0	11 (73)
	ECOG PS 1	4 (27)
**Caregiver**		
	Spouse	13 (87)
	Other^c^	2 (13)
**Melphalan dose (mg/m** ^2^ **)**		
	200	12 (80)
	140	3 (20)

^a^SCT: stem cell transplantation.

^b^ECOG PS: Eastern Cooperative Oncology Group performance status.

^c^Other caregivers included an ex-spouse for one patient and a sibling for another patient.

### Feasibility of the DLC Platform

**Figure 2:**
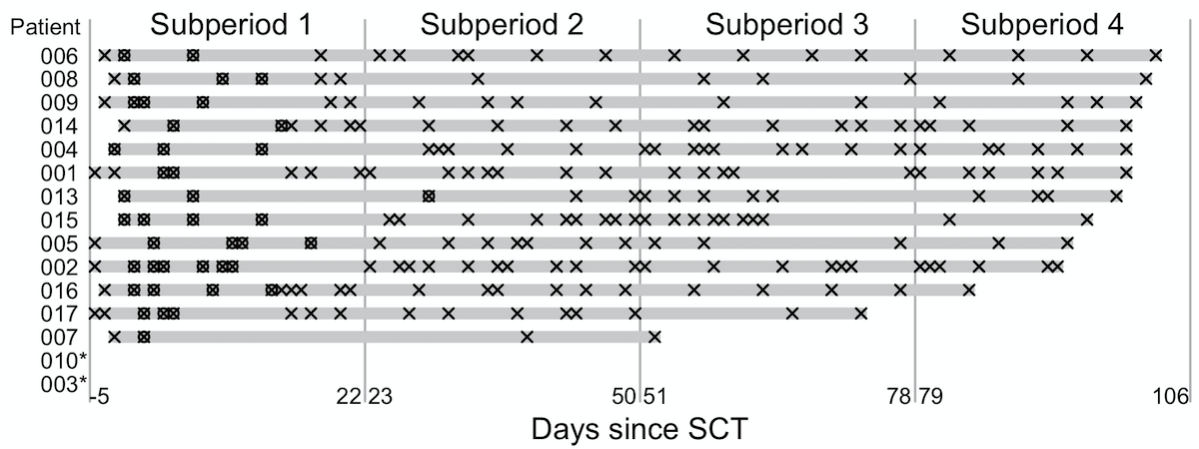
Timeplot of bidirectional conversations. Days relative to SCT during which at least one bidirectional conversation took place are marked either by unboxed X icons (for outpatient) or boxed X icons (for inpatient). The 16-week study subperiod is divided into four 28-day subperiods as shown. *These patients underwent SCT but dropped out of the study prior to digital life coaching initiation. SCT: stem cell transplantation.

Bidirectional conversations during the 16-week study period are depicted in Figure 2. Of the 15 enrolled patients who underwent SCT, 11 (73%) met our primary endpoint of ongoing engagement. Of the remaining 4/15 (27%) patients, 2 (50%) dropped out of the study prior to DLC initiation while an additional 2 (50%) demonstrated ongoing engagement only for the first 3 of the 4 study subperiods. For the 13/15 (87%) patients who received any coaching, the median number of conversation-days (defined as discrete days with at least one bidirectional conversation) during the 16-week study period was 18 (range 4-29). This corresponded to a median engagement frequency of 1 conversation every 6.2 days (range 3.9-28 days). During inpatient hospitalizations, this corresponding frequency was 1 conversation every 5.3 days (range 2.7-15 days). Of 240 conversation-days across 13 patients, 120 (50%) occurred via SMS text messaging while 109 (45%) occurred exclusively via phone calls. Of note, 69% (9/13) of patients never used emails to engage with their coaches.

We did not formally request feedback from patients about the DLC curriculum with respect to its feasibility or overlap with existing clinical resources. However, 2/13 (15%) patients did opt to reply at least once to automated ePRO assessment emails (correspondences that were then forwarded to the study team nonurgently). One patient wrote that her coach was “fantastic for answering questions, hearing and airing concerns…and mostly boosting hope, which is so very necessary in the MM world.” A second patient’s caregiver responded to an automated ePRO prompt inquiring about any additional medications for the management of neuropathy; however, whether this question had been redirected to the study team by the patient’s coach was not specified.

### Longitudinal Patient-Reported Outcomes

**Figure 3:**
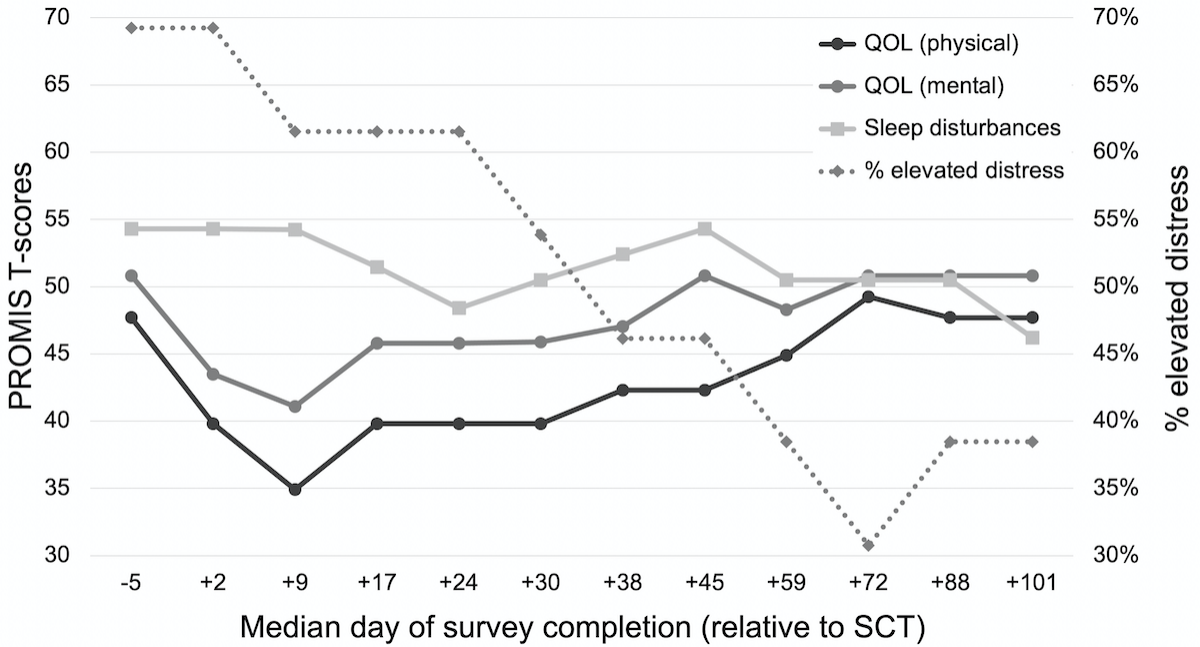
Repeated ePRO assessments over time. For QOL (physical), QOL (mental), and sleep disturbances, values represent population-adjusted T-scores with a mean of 50 and SD of 10. Higher values represent better physical/mental QOL and worsened sleep, respectively. For % elevated distress, values represent the percentage of patients at each timepoint who reported elevated distress (defined as a Distress Thermometer score of 4 or higher) [58]. ePRO: electronic patient-reported outcome; PROMIS: Patient-Reported Outcome Measurement Information System; QOL: quality of life; SCT: stem cell transplantation.

The results of ePRO assessments for the 13/15 (87%) patients who received any coaching are depicted in Figure 3. A total of 94% (147/156) of ePRO assessments were completed, with a mean time of 3.3 minutes (range 1.1-17.9 minutes) spent per 15-question assessment. The results of these assessments were not shared with patients’ coaches or clinical teams at any point. Compared to baseline values assessed at a median of day −5, both the physical and mental components of QOL nadired during the second week after SCT (median day +9). There were no clinically meaningful exacerbations in sleep disturbances during the study period when compared to baseline. A total of 69% (9/13) of patients exhibited elevated distress at baseline (median day −5) and at the second timepoint (median day +2); this percentage decreased steadily in subsequent weeks to a nadir of 31% (4/13) in the eleventh week (median day +72).

## Discussion

### Main Findings

In our single-arm pilot study, we found that DLC can be feasible for selected patients with MM during the intensive 100-day period encompassing autologous SCT. Although our study enrolled patients immediately prior to 2-week hospitalizations for myeloablative chemotherapy, the rate of ongoing patient engagement of 73% over a 3-month period was similar to or approached that of DLC-type interventions designed for ambulatory survivors beyond day +100 after SCT [41,42]. Hospitalized patients who underwent SCT in our study engaged bidirectionally with their coaches approximately once per week on average, a frequency comparable to that of unidirectional mobile health apps for patient education or ePRO completion [59,60]. Based on the positive results of this pilot study, a randomized phase 2 study of DLC versus usual care in this population is underway to investigate the efficacy of DLC (ClinicalTrials.gov NCT04589286).

One lesson learned from our study was the optimal timing of supportive interventions during intensive cancer-directed treatments. Our DLC intervention began on day −5 of SCT, which preceded hospital admission for high-dose chemotherapy by 2-3 days. Our rationale for this time frame was to focus on the acute posttransplant period itself, a time during which QOL is known to decrease because of increased symptom burden and acute life disruptions [11-15]. However, in contrast to 2 prior studies that demonstrated that distress peaked at count nadir or hospital discharge [3,15], this study found that elevated distress was highest at pre-SCT baseline. As a second observation of note, our narrow pre-SCT window also precluded participation for 1 of our 15 enrolled patients who was unable to connect with her coach before hospitalization. Earlier initiation of DLC, as we have implemented in our ongoing phase 2 study, may allow coaches to intervene during emotional distress when this symptom is at its peak while also improving the logistical adoptability of DLC for patients.

A second lesson learned from our study was the importance of flexibility and bidirectionality regarding how patients can engage with coaches. Our DLC platform allowed patients to call, text, or email their coaches in the same ways in which they might communicate with loved ones. All communications sent by coaches were intentionally worded to encourage subsequent responses from patients. Prior research has suggested that the promotion of interactivity may improve patients’ perceptions of and patient engagement with digital platforms [61,62]. Although we did not formally test this hypothesis, these characteristics may have enhanced the “stickiness” of DLC (defined as the ease of continued use of a patient-facing platform over time) [63] during the peri-SCT period. As a counterexample within digital oncology, the randomized Southwest Oncology Group S1105 study of automated twice-weekly unidirectional SMS text messaging for women with breast cancer did not show a benefit in its primary endpoint of medication adherence [64]. The authors posited that their approach may not have sufficiently engaged patients to promote behavioral change.

### Limitations and Future Directions

Our study nevertheless had several limitations; the most important one was the limited external validity from our small cohort of English-speaking patients who owned personal mobile phones. Although our intervention was deemed acceptable by patients who were planning to undergo SCT (with 85% of approached patients enrolling in the study), our patient population was relatively homogenous with regard to race, ethnicity, and marital status. Because racial and socioeconomic barriers affect SCT access and post-SCT outcomes in patients with MM [65,66], further investigation of this platform in a broader population of patients is needed. Two active areas of expansion for the DLC vendor include translation of content into other languages and development of an entirely landline-based curriculum (eg, using hospital-based phones and house-based phones) for patients who do not personally own mobile phones.

Other limitations of our study include our inability to isolate the specific coaching frameworks or themes that were most useful for patients. While patients communicated with their coaches approximately once per week on average, we did not directly gauge patient rationales for DLC engagement at each timepoint. As such, it is possible that patients may have maintained interactions with their coaches out of politeness rather than firm beliefs that the coaching was of value. Elucidating the specific impact of DLC on ePRO assessments of QOL is thus a key goal of our ongoing randomized phase 2 study. Furthermore, our ePRO results must be interpreted with caution given the small sample size and the presence of DLC itself as a potential confounder. Our finding that elevated distress was most prevalent in the days immediately before and after SCT was predicated on scores of 4 or higher on the single-question NCCN Distress Thermometer, a definition used previously in the SCT population [67,68]. It is unclear whether the use of longer survey-based instruments to assess distress, such as the Brief Symptom Inventory or Impact of Event Scale, would have yielded different results.

While DLC-type interventions have already been investigated in ambulatory cancer survivors [32,33,36-39,41,42], this is the first study to our knowledge to investigate DLC during a hospital-based cancer therapy such as SCT. DLC offers 3 possible benefits over traditional face-to-face tools during such intensive treatment modalities. First, although interventions with substantial in-person components may be more likely to improve distress in patients with cancer [69], additional in-person visits may be impractical for patients to attend in the setting of acute symptomatic toxicities. In contrast, DLC allows patients to access a centralized team of life coaches from the convenience of their phones regardless of their current location. Second, for patients who interact frequently with their coaches, “micro-learning” (a key functionality of mobile health tools) [70] may enhance the staying power of the coaching curriculum. Third, as discussed previously, DLC can readily accommodate individual patient preferences with regard to specific communication modalities and cadences.

However, whether DLC can truly improve the quality of supportive care during SCT requires further investigation in our ongoing randomized phase 2 trial and subsequent investigations. Similarly, given a plethora of ePRO instruments used to assess QOL and distress in the hematopoietic stem cell transplant population [71], the PROMIS Global Health Scale and NCCN Distress Thermometer—both of which are relatively newer in this patient population—require further validation against longer survey-based instruments such as the Functional Assessment of Cancer Therapy Bone Marrow Transplantation or Brief Symptom Inventory assessments. Expansion of DLC into other transplantation settings, particularly for patients with acute leukemia undergoing allogeneic SCT, is warranted given that patients who undergo allogeneic SCT have a higher symptom burden than those who undergo autologous SCT [11,72]. This is an active area of investigation for our group.

### Conclusions

Selected patients receiving high-dose chemotherapy followed by autologous SCT can engage meaningfully with life coaches using their phones, even during 2-week hospitalizations. A randomized phase 2 study to assess the efficacy of DLC in this population is underway. If future studies demonstrate the effectiveness of DLC in improving QOL and symptom burden during SCT, this type of intervention may eventually become a routine tool for supporting patient well-being during intensive cancer-directed therapies.

## Abbreviations

DLC: digital life coaching
ePRO: Electronic patient-reported outcomes
MM: multiple myeloma
NCCN: National Comprehensive Cancer Network
PROMIS: Patient-reported Outcome Measurement Information System
QOL: quality of life
REDCap: Research Electronic Data Capture
SCT: stem cell transplantation
SMART: Specific, Measurable, Achievable, Relevant, and Time-based
